# Association of Upfront Peptide Receptor Radionuclide Therapy With Progression-Free Survival Among Patients With Enteropancreatic Neuroendocrine Tumors

**DOI:** 10.1001/jamanetworkopen.2022.0290

**Published:** 2022-02-24

**Authors:** Sara Pusceddu, Natalie Prinzi, Salvatore Tafuto, Toni Ibrahim, Angelina Filice, Maria Pia Brizzi, Francesco Panzuto, Sergio Baldari, Chiara M. Grana, Davide Campana, Maria Vittoria Davì, Dario Giuffrida, Maria Chiara Zatelli, Stefano Partelli, Paola Razzore, Riccardo Marconcini, Sara Massironi, Fabio Gelsomino, Antongiulio Faggiano, Elisa Giannetta, Emilio Bajetta, Franco Grimaldi, Mauro Cives, Fernando Cirillo, Vittorio Perfetti, Francesca Corti, Claudio Ricci, Luca Giacomelli, Luca Porcu, Massimo Di Maio, Ettore Seregni, Marco Maccauro, Secondo Lastoria, Alberto Bongiovanni, Annibale Versari, Irene Persano, Maria Rinzivillo, Salvatore Antonio Pignata, Paola Anna Rocca, Giuseppe Lamberti, Sara Cingarlini, Ivana Puliafito, Maria Rosaria Ambrosio, Isabella Zanata, Alessandra Bracigliano, Stefano Severi, Francesca Spada, Valentina Andreasi, Roberta Modica, Federica Scalorbi, Massimo Milione, Giovanna Sabella, Jorgelina Coppa, Riccardo Casadei, Maria Di Bartolomeo, Massimo Falconi, Filippo de Braud

**Affiliations:** 1 Department of Medical Oncology, Fondazione IRCCS Istituto Nazionale dei Tumori di Milano, European Neuroendocrine Tumor Society (ENETS) Center of Excellence, Milan, Italy; 2 Oncologia Clinica e Sperimentale Sarcomi e Tumori Rari, Istituto Nazionale Tumori IRCCS, Fondazione G. Pascale, Naples, Italy; 3 Osteoncology and Rare Tumors Center, IRCCS Istituto Romagnolo per lo Studio dei Tumori “Dino Amadori,” Meldola, Italy; 4 Nuclear Medicine Unit, Azienda Unità Sanitaria Locale–IRCCS di Reggio Emilia, Reggio Emilia, Italy; 5 Azienda Ospedaliera Universitaria San Luigi Gonzaga, Orbassano, Italy; 6 Digestive Disease Unit, Sant’Andrea University Hospital, ENETS Center of Excellence, Rome, Italy; 7 Department of Biomedical and Dental Sciences and Morphofunctional Imaging, Nuclear Medicine Unit, University of Messina, Messina, Italy; 8 Division of Nuclear Medicine, IRCCS Istituto Europeo di Oncologia, Milan, Italy; 9 Department of Experimental Diagnostic and Specialized Medicine, Alma Mater Studiorum, University of Bologna, Bologna, Italy; 10 Division of Medical Oncology, IRCCS Azienda Ospedaliera–Universitaria Bologna, Neuroendocrine Tumor Team Bologna, ENETS Center of Excellence Bologna, Bologna, Italy; 11 Department of Medicine, Section of Endocrinology, University and Hospital Trust of Verona, ENETS Center of Excellence, Verona, Italy; 12 Oncologia Medica, Istituto Oncologico del Mediterraneo, Viagrande (Catania), Italy; 13 Department of Medical Sciences, Section of Endocrinology, Geriatrics and Internal Medicine, University of Ferrara, Ferrara, Italy; 14 Pancreatic Surgery, Pancreas Translational and Clinical Research Center, San Raffaele Hospital IRCCS, Università Vita-Salute San Raffaele, ENETS Center of Excellence, Milano, Italy; 15 Department of Internal Medicine, Division of Endocrinology, A.O. Ordine Mauriziano, Turin, Italy; 16 Department of Oncology, Santa Chiara Hospital, Azienda Ospedaliero–Universitaria Pisana, Pisa, Italy; 17 Division of Gastroenterology, Ospedale San Gerardo, University of Milano–Bicocca, Monza, Italy; 18 Department of Oncology and Haematology, University Hospital of Modena, Modena, Italy; 19 Department of Clinical and Molecular Medicine, Endocrinology Unit, Sant’Andrea Hospital, Sapienza University of Rome, Rome, Italy; 20 Department of Experimental Medicine, Sapienza Università Roma, Rome, Italy; 21 Istituto di Oncologia, Policlinico di Monza, Monza, Italy; 22 Endocrinology and Metabolism Unit, University Hospital S. Maria della Misericordia, Udine, Italy; 23 Department of Biomedical Sciences and Human Oncology, University of Bari, Bari, Italy; 24 National Cancer Center, Tumori Institute Giovanni Paolo II, Bari, Italy; 25 Department of Surgery, General Surgery Unit, Gruppo Tumori Rari, Azienda Socio-Sanitaria Territoriale–Cremona, Cremona, Italy; 26 ASST–Pavia Medicina Interna Varzi, Varzi, Italy; 27 Division of Pancreatic Surgery, IRCCS Azienda Ospedaliero–Universitaria Di Bologna, Bologna, Italy; 28 Department of Internal Medicine and Surgery, Alma Mater Studiorum, University of Bologna, Bologna, Italy; 29 Polistudium, Milan, Italy; 30 Methodology for Clinical Research Laboratory, Oncology Department, IRCCS Istituto di Ricerche Farmacologiche Mario Negri, Milan, Italy; 31 Department of Oncology, University of Turin, A.O. Ordine Mauriziano, Torino, Italy; 32 Department of Nuclear Medicine, Fondazione IRCCS Istituto Nazionale dei Tumori di Milano, ENETS Center of Excellence, Milan, Italy; 33 Nuclear Medicine Unit, Istituto Nazionale Tumori IRCCS, Fondazione G. Pascale, Naples, Italy; 34 Nuclear Medicine Unit, Azienda Ospedaliera Papardo, Messina, Italy; 35 Department of Medicine, Oncology, University and Hospital Trust of Verona, ENETS Center of Excellence, Verona, Italy; 36 Nuclear Medicine Therapy Unit, IRCCS Istituto Romagnolo per lo Studio dei Tumori “Dino Amadori,” Meldola, Italy; 37 Division of Medical Oncology, IRCCS Istituto Europeo di Oncologia, Milan, Italy; 38 Department of Clinical Medicine and Surgery, University of Naples Federico II, Naples, Italy; 39 First Division of Pathology, Department of Pathology and Laboratory Medicine, IRCCS Foundation National Cancer Institute, Milan, Italy; 40 Gastro-entero-pancreatic Surgical and Liver Transplantation Unit, Fondazione IRCCS Istituto Nazionale dei Tumori di Milano, ENETS Center of Excellence, Milan, Italy; 41 Department of Oncology and Hemato-Oncology, Università deli Studi di Milano, Milan, Italy

## Abstract

**Importance:**

Data about the optimal timing for the initiation of peptide receptor radionuclide therapy (PRRT) for advanced, well-differentiated enteropancreatic neuroendocrine tumors are lacking.

**Objective:**

To evaluate the association of upfront PRRT vs upfront chemotherapy or targeted therapy with progression-free survival (PFS) among patients with advanced enteropancreatic neuroendocrine tumors who experienced disease progression after treatment with somatostatin analogues (SSAs).

**Design, Setting, and Participants:**

This retrospective, multicenter cohort study analyzed the clinical records from 25 Italian oncology centers for patients aged 18 years or older who had unresectable, locally advanced or metastatic, well-differentiated, grades 1 to 3 enteropancreatic neuroendocrine tumors and received either PRRT or chemotherapy or targeted therapy after experiencing disease progression after treatment with SSAs between January 24, 2000, and July 1, 2020. Propensity score matching was done to minimize the selection bias.

**Exposures:**

Upfront PRRT or upfront chemotherapy or targeted therapy.

**Main Outcomes and Measures:**

The main outcome was the difference in PFS among patients who received upfront PRRT vs among those who received upfront chemotherapy or targeted therapy. A secondary outcome was the difference in overall survival between these groups. Hazard ratios (HRs) were fitted in a multivariable Cox proportional hazards regression model to adjust for relevant factors associated with PFS and were corrected for interaction with these factors.

**Results:**

Of 508 evaluated patients (mean ([SD] age, 55.7 [0.5] years; 278 [54.7%] were male), 329 (64.8%) received upfront PRRT and 179 (35.2%) received upfront chemotherapy or targeted therapy. The matched group included 222 patients (124 [55.9%] male; mean [SD] age, 56.1 [0.8] years), with 111 in each treatment group. Median PFS was longer in the PRRT group than in the chemotherapy or targeted therapy group in the unmatched (2.5 years [95% CI, 2.3-3.0 years] vs 0.7 years [95% CI, 0.5-1.0 years]; HR, 0.35 [95% CI, 0.28-0.44; *P* < .001]) and matched (2.2 years [95% CI, 1.8-2.8 years] vs 0.6 years [95% CI, 0.4-1.0 years]; HR, 0.37 [95% CI, 0.27-0.51; *P* < .001]) populations. No significant differences were shown in median overall survival between the PRRT and chemotherapy or targeted therapy groups in the unmatched (12.0 years [95% CI, 10.7-14.1 years] vs 11.6 years [95% CI, 9.1-13.4 years]; HR, 0.81 [95% CI, 0.62-1.06; *P* = .11]) and matched (12.2 years [95% CI, 9.1-14.2 years] vs 11.5 years [95% CI, 9.2-17.9 years]; HR, 0.83 [95% CI, 0.56-1.24; *P* = .36]) populations. The use of upfront PRRT was independently associated with improved PFS (HR, 0.37; 95% CI, 0.26-0.51; *P* < .001) in multivariable analysis. After adjustment of values for interaction, upfront PRRT was associated with longer PFS regardless of tumor functional status (functioning: adjusted HR [aHR], 0.39 [95% CI, 0.27-0.57]; nonfunctioning: aHR, 0.29 [95% CI, 0.16-0.56]), grade of 1 to 2 (grade 1: aHR, 0.21 [95% CI, 0.12-0.34]; grade 2: aHR, 0.52 [95% CI, 0.29-0.73]), and site of tumor origin (pancreatic: aHR, 0.41 [95% CI, 0.24-0.61]; intestinal: aHR, 0.19 [95% CI, 0.11-0.43]) (*P* < .001 for all). Conversely, the advantage was not retained in grade 3 tumors (aHR, 0.31; 95% CI, 0.12-1.37; *P* = .13) or in tumors with a Ki-67 proliferation index greater than 10% (aHR, 0.73; 95% CI, 0.29-1.43; *P* = .31).

**Conclusions and Relevance:**

In this cohort study, treatment with upfront PRRT in patients with enteropancreatic neuroendocrine tumors who had experienced disease progression with SSA treatment was associated with significantly improved survival outcomes compared with upfront chemotherapy or targeted therapy. Further research is needed to investigate the correct strategy, timing, and optimal specific sequence of these therapeutic options.

## Key Points

**Question:**

Is upfront peptide receptor radionuclide therapy (PRRT) associated with improved progression-free survival (PFS) compared with upfront chemotherapy or targeted therapy in patients with enteropancreatic neuroendocrine tumors who experienced disease progression after somatostatin analogue treatment?

**Findings:**

In this cohort study of 508 patients with enteropancreatic neuroendocrine tumors, the use of upfront PRRT immediately after disease progression with somatostatin analogue treatment was associated with significantly improved progression-free survival compared with upfront chemotherapy or targeted therapy.

**Meaning:**

The findings suggest that upfront PRRT in patients with enteropancreatic neuroendocrine tumors who experienced disease progression with SSA treatment may be associated with significantly longer progression-free survival compared with chemotherapy or targeted therapy.

## Teaser

This cohort study assesses the association of upfront peptide receptor radionuclide therapy (PRRT) vs upfront chemotherapy or targeted therapy with progression-free survival among patients in Italy with enteropancreatic neuroendocrine tumors who experienced disease progression after treatment with somatostatin analogues.

## Introduction

The prevalence and incidence of gastroenteropancreatic neuroendocrine tumors have been increasing. The Surveillance, Epidemiology, and End Results database reported a 20-year prevalence of 48 gastroenteropancreatic neuroendocrine tumors per 100 000 population and an incidence of 3.56 per 100 000 population per year in the US.^1^ Compared with the aggressive course of high-grade neuroendocrine carcinomas, the natural evolution of well-differentiated gastroenteropancreatic neuroendocrine tumors is usually indolent, with a median overall survival (OS) of 14 to 30 years among patients with localized, radically resected forms.^1^ However, approximately 50% of patients with gastroenteropancreatic neuroendocrine tumors have advanced disease at diagnosis.^2^ For these patients, the prognosis is poor, with a median OS ranging from only 4 months to 6 years.^1^

For patients with localized gastroenteropancreatic neuroendocrine tumors, curative surgery is the gold standard, whereas in patients with unresectable neuroendocrine tumors, the treatment goal is to prolong survival, improve and maintain quality of life, and control tumor growth and secretory symptoms. Somatostatin analogues (SSAs), chemotherapy, sunitinib, everolimus, and peptide receptor radionuclide therapy (PRRT) have become mainstays of treatment in patients with low- or intermediate-grade gastroenteropancreatic neuroendocrine tumors.^3,4,5,6,7,8^

Studies have shown that PRRT was effective for advanced gastroenteropancreatic neuroendocrine tumors, especially in patients with high somatostatin receptor expression.^7,9,10^ In the NETTER-1 study, the only randomized, phase 3 trial of patients with inoperable, somatostatin receptor–positive midgut carcinoid tumors conducted to date, treatment with lutetium 177 (^177^Lu)–dotatate significantly improved progression-free survival (PFS) and tumor response compared with high-dose long-acting octreotide.^7^

However, although PRRT with ^177^Lu-dotatate was approved by the European Medicines Agency^11^ and the US Food and Drug Administration^12^ for the treatment of patients with unresectable, low- or intermediate-grade, locally advanced or metastatic gastroenteropancreatic neuroendocrine tumors, the NETTER-1 trial proved the safety and efficacy of PRRT only for well-differentiated midgut neuroendocrine tumors as second-line therapy after disease progression in patients who had received SSA treatment.^7^ Data on PRRT in pancreatic neuroendocrine tumors or concerning the proper timing for the initiation of PRRT and its effectiveness in comparison with chemotherapy or targeted therapy for neuroendocrine tumors are lacking.

Therefore, especially for advanced, well-differentiated pancreatic neuroendocrine tumors, targeted therapy and chemotherapy still represent treatments of choice, and the use of PRRT has been recommended only after failure of these therapies.^13,14,15,16,17,18^ Guidelines from the European Society for Medical Oncology,^13^ European Neuroendocrine Tumor Society,^16,17,18^ North American Neuroendocrine Tumor Society,^14^ and National Comprehensive Cancer Network^15^ outline chemotherapy as the primary treatment; however, physicians’ practice may vary (SSAs and/or chemotherapy and/or everolimus or sunitinib used as first- and/or second-line treatment), and the use of these agents as first- or second-line treatment is still individualized. The identification of the correct timing and sequence of PRRT for well-differentiated enteropancreatic neuroendocrine tumors represents an important unmet medical need.

We performed a multicenter, retrospective cohort study to evaluate the association of the early use of PRRT vs other treatment options (chemotherapy or targeted therapy) with survival outcomes (PFS and OS) in a large population of patients in Italy with advanced enteropancreatic neuroendocrine tumors who experienced disease progression after SSA therapy.

## Methods

### Study Setting

This was a multicenter, retrospective cohort study of patients with advanced enteropancreatic neuroendocrine tumors who received PRRT or chemotherapy or targeted therapy as second-line treatment after disease progression with SSA treatment between January 24, 2000, and July 1, 2020, at 25 Italian oncology centers. The study was conducted according to the Declaration of Helsinki^19^ and was approved by the institutional review board of the coordinating center, Fondazione IRCCS Istituto Nazionale dei Tumori di Milano, in Milan, Italy. All patients provided written informed consent for the use of their data for research purposes. The study followed the Strengthening the Reporting of Observational Studies in Epidemiology (STROBE) reporting guideline.

Patients aged 18 years or older were eligible if they had an unresectable, locally advanced or metastatic, well-differentiated enteropancreatic neuroendocrine tumor classified as grades 1 to 3 according to the 2019 World Health Organization (WHO) classification system.^20^ Other eligibility criteria were (1) adequate tracer accumulation on somatostatin receptor imaging (^68^Ga-DOTATOC positron emission tomography/computed tomography [PET/CT] images or Octreoscan); (2) disease documented as progressive according to the Response Evaluation Criteria in Solid Tumors (RECIST), version 1.1, based on CT or magnetic resonance imaging after first-line treatment with SSAs (octreotide long-acting release, 20-30 mg, every 3-4 weeks, or lanreotide autogel, 120 mg, every 3-4 weeks) at baseline or within 12 months before baseline; and (3) second-line treatment with PRRT (yttrium 90, lutetium 177, or both), PRRT and SSAs, or targeted therapy (everolimus or sunitinib) or chemotherapy (temozolomide, cisplatin, oxaliplatin, or fluorouracil) alone or in combination with SSAs. Patients were ineligible if they had a poorly differentiated neuroendocrine carcinoma or had not received at least 2 cycles of PRRT.

### Objectives and Study Design

The primary objective of this study was to investigate differences in PFS between patients who received upfront PRRT vs patients who received upfront chemotherapy or targeted therapy after experiencing disease progression with SSA treatment. Secondary objectives were to evaluate differences in OS between patients who received upfront PRRT vs upfront chemotherapy or targeted therapy and differences in PFS and OS between patients who received upfront PRRT vs upfront chemotherapy or targeted therapy in prespecified patient subgroups (pancreatic vs intestinal neuroendocrine tumor, neuroendocrine tumor with a Ki-67 proliferation index greater than 10% vs 10% or less according to the 2019 WHO classification,^20^ and functioning vs nonfunctioning tumors). Survival analyses were performed in an unmatched population and a propensity score–matched population to minimize the risk of selection bias.

Progression-free survival was defined as the time from second-line treatment initiation (PRRT or chemotherapy or targeted therapy) to disease progression (assessed according to each center’s clinical practice) or death from any cause. To avoid immortal time bias in the survival analysis, OS was defined as the time from diagnosis of locally advanced or metastatic disease to death from any cause. Patients lost to follow-up were censored at the last visit. Curves of disease progression and overall mortality were computed using the Kaplan-Meier method. The log-rank test was used to compare survival functions.

Disease progression was measured according to RECIST, version 1.1, based on a set of measurable lesions identified as target lesions at the baseline of treatment and followed up until disease progression, together with other lesions denoted as nontarget lesions. Tumor radiological assessment (CT or magnetic resonance imaging, ^68^Ga-DOTATOC PET/CT or Octreoscan) was performed at diagnosis, before treatment initiation, and during treatment. There were no predefined time points for the radiological assessment of target and nontarget lesions during treatment. However, before initiation of PRRT or chemotherapy or targeted therapy, all patients were required to undergo radiological assessments after a confirmatory status of disease progression according to RECIST, version 1.1 (assessed by the local center by CT or magnetic resonance imaging) at baseline or within 12 months before baseline. During treatment, the radiological assessment was repeated every 3 to 4 months for most patients.

### Statistical Analysis

Patient characteristics were analyzed by descriptive statistics. Fisher exact and χ^2^ tests were used for categorical variables; continuous variables were analyzed using the *t* test.

Propensity score matching was performed to minimize selection bias owing to the retrospective design. For matching, we used the following variables: time to disease progression during SSA therapy, sex, age, Eastern Cooperative Oncology Group performance status, tumor origin, tumor function, the presence of multiple endocrine neoplasia type 1 syndrome, WHO grade, Ki-67 proliferation index with a 10% cutoff, surgery for primary tumors, synchronous or metachronous metastases, surgery for metastases, number and type of metastatic sites, locoregional therapy, sequence (PRRT followed by chemotherapy or targeted therapy or chemotherapy or targeted therapy followed by PRRT), type of SSA during PRRT, number of cycles of PRRT, and type of radionuclide. The matching was created with a 1:1 ratio and was made using the neighborhood method with a caliper width of 0.05 pooled SDs. For each variable, the effect of the selection bias was measured as standardized bias before and after matching. A standardized bias value less than 15% indicates an optimal balance. We also reported the standardized mean difference (SMD) to assess the balance between the 2 groups. An SMD of 0.2 or less indicates that the percentage of nonoverlapped population was 15% or less; greater than 0.2 to 0.5, the percentage of nonoverlapped population was 33% or less; greater than 0.5 to 0.8, the percentage of nonoverlapped population was 50% or less; and greater than 0.8, the percentage of nonoverlapped population was greater than 50%. All endpoints were reported for the unmatched and matched populations.

A survival analysis was performed using a semiparametric approach (Cox proportional hazards regression); medians, hazard ratios (HRs), and 95% CIs are reported. The HRs for PFS in the treatment groups were fitted in a multivariable Cox proportional hazards regression model along with the most relevant known factors associated with PFS (functioning tumors, primary site, WHO grade, and Ki-67 proliferation index cutoff of 10%). Moreover, the HR values of both groups were adjusted for the interaction with these factors.

All statistical tests were 2-tailed, and *P* < .05 was considered significant. Statistical analyses were performed using Stata, version 16 (StataCorp LLC).

## Results

### Patient Characteristics of Unmatched and Matched Groups

We screened 618 records from a consecutive sample; 110 patients were excluded owing to not meeting inclusion criteria (eFigure 1 in the Supplement). In the final analysis, 508 patients were included (mean ([SD] age, 55.7 [0.5] years; 278 [54.7%] were male), of whom 179 (35.2%) received upfront chemotherapy or targeted therapy and 329 (64.8%) received upfront PRRT. In Italy, targeted therapy was not approved for use in clinical practice until 2011; in the group receiving upfront chemotherapy or targeted therapy, 50 patients (27.9%) experienced disease progression before 2011; in the group receiving upfront PRRT, 92 patients (28.0%) experienced disease progression before 2011.

Table 1 and the eTable in the Supplement show demographic, clinical, and pathological characteristics of the unmatched population. The 2 groups did not differ with regard to time to disease progression during SSA therapy, sex, age, Eastern Cooperative Oncology Group performance status, presence of multiple endocrine neoplasia type 1 syndrome, rate of synchronous metastases, surgery for metastases, number of metastases, site of metastases, locoregional treatment, and SSA therapy rate during PRRT.

**Table 1.** Baseline Characteristics of Unmatched Populations of Patients With Enteropancreatic Neuroendocrine Tumors Who Received Upfront PRRT or Upfront Chemotherapy or Targeted Therapy

Variable	Patients^a^			*P* value^b^	SMD^c^	Standardized bias, %^d^
	Total (N = 508)	Chemotherapy or targeted therapy (n = 179)	PRRT (n = 329)			
Time to progression during first-line SSA therapy, mean (SD), mo	26.9 (3.1)	32.7 (8.1)	23.8 (1.7)	.16	0.12	64.1
Sex						
Female	230 (45.3)	79 (44.1)	151 (45.9)	.38	0.03	3.4
Male	278 (54.7)	100 (55.9)	178 (54.1)			
Age, mean (SD), y	55.7 (0.5)	55.3 (0.9)	55.9 (0.6)	.51	0.06	6.3
ECOG performance status						
≤1	478 (94.1)	167 (93.3)	311 (94.5)	.50	0.10	5.1
>2	25 (4.9)	11 (6.1)	14 (4.3)			
Data missing	5 (1.0)	1 (0.6)	4 (1.2)			
Site of tumor origin						
Pancreas	260 (51.2)	137 (76.5)	123 (37.4)	<.001	0.84	86.4
Intestine	248 (48.8)	42 (23.5)	206 (62.6)			
Functioning						
No	162 (31.8)	42 (23.5)	120 (36.5)	.008	0.29	30.4
Yes	345 (67.9)	137 (76.5)	208 (63.2)			
Data missing	1 (0.2)	0	1 (0.3)			
MEN1 syndrome						
No	502 (98.8)	178 (99.4)	324 (98.5)	.67	0.08	10.3
Yes	6 (1.2)	1 (0.6)	5 (1.5)			
Grade according to 2019 WHO classificiation^20^						
1	203 (40.0)	49 (27.4)	154 (46.8)	<.001	0.38	85.3
2	272 (53.5)	112 (62.6)	160 (48.6)			
3	15 (2.9)	11 (6.1)	4 (1.2)			
Data missing	18 (3.5)	7 (3.9)	11 (3.3)			
Ki-67 proliferation index >10%						
No	408 (80.3)	125 (69.8)	283 (86.0)	<.001	0.33	40.3
Yes	78 (15.4)	45 (25.2)	33 (10.0)			
Data missing	22 (4.3)	9 (5.0)	13 (4.0)			
Surgery for primary tumor						
No	171 (33.7)	83 (46.4)	88 (26.8)	<.001	0.47	45.4
Yes	337 (66.3)	96 (53.6)	241 (73.3)			
Metastases						
Synchronous	432 (85.0)	152 (84.9)	280 (85.1)	>.99	0.01	3.9
Metachronous	76 (15.0)	27 (15.1)	49 (14.9)			
Surgery for metastases						
No	360 (70.9)	128 (71.5)	232 (70.5)	.82	0.13	4.1
Yes	146 (28.7)	51 (28.5)	95 (28.8)			
Data missing	2 (0.4)	0	2 (0.6)			
Metastatic sites, No.						
1	186 (36.6)	67 (37.4)	119 (36.2)	.22	0.04	14.8
2	179 (35.2)	67 (37.4)	112 (34)			
≥3	117 (23.1)	33 (18.4)	84 (25.5)			
Data missing	26 (5.1)	12 (6.7)	14 (4.3)			
Metastatic sites						
Not reported	26 (5.1)	12 (6.7)	14 (4.3)	.58	0.11	9.8
Liver	166 (32.7)	61 (34.1)	105 (31.9)			
Liver and extrahepatic	288 (56.7)	97 (54.2)	191 (58.1)			
Extrahepatic	28 (5.5)	9 (5.0)	19 (5.8)			
Hepatic locoregional treatment						
No	375 (73.8)	128 (71.5)	247 (75.1)	.39	0.08	6.6
Yes	133 (26.2)	51 (28.5)	82 (24.9)			
Sequence completed^e^						
No	12 (2.4)	0	12 (3.7)	.01	0	0
Yes	496 (97.6)	179 (100)	317 (96.3)			
SSA during PRRT						
No	62 (12.2)	24 (13.4)	38 (11.6)	.50	0.08	6.3
Standard	443 (87.2)	155 (86.6)	288 (87.5)			
High dose	3 (0.6)	0	3 (0.9)			
Cycles of PRRT, mean (SD), No.	4.7 (0.1)	4.2 (0.1)	5.0 (0.1)	<.001	0.49	50.8
Radionuclide						
Not reported	12 (2.4)	1 (0.6)	11 (3.3)	.007	0.40	16.3
Yttrium-90	115 (22.6)	54 (30.2)	61 (18.5)			
Lutetium-177	224 (44.1)	75 (41.9)	149 (45.3)			
Both	157 (30.9)	49 (27.4)	108 (2.8)			

Abbreviations: ECOG, Eastern Cooperative Oncology Group; MEN1, multiple endocrine neoplasm type 1; PRRT, peptide receptor radionuclide therapy; SMD, standardized mean difference; SSA, somatostatin analogue; WHO, World Health Organization.

^a^ Data are reported as the number (percentage) of patients unless otherwise indicated.

^b^ The Fisher exact test was used for binary variables, the Pearson χ^2^ test for ordinal variables, and the Student *t* test for continuous variables.

^c^ Effect size categories: small, 0 to 0.2 (nonoverlap population <15%); medium, greater than 0.2 to 0.5 (nonoverlap population <33%); large, greater than 0.5 to 0.8 (nonoverlap population <50%); and very large, greater than 0.8 (nonoverlap population >50%).

^d^ Standardized bias reflects the selection bias as a percentage; a value less than 15% indicated an optimal balance.

^e^ In the upfront chemotherapy or targeted therapy group, the sequence was considered completed when PRRT was used after chemotherapy or targeted therapy failure; in the upfront PRRT group, the sequence was considered completed when chemotherapy or targeted therapy was used after PRRT failure.

Compared with patients who received upfront PRRT, those who received upfront chemotherapy or targeted therapy more frequently had neuroendocrine tumors of pancreatic origin (137 patients [76.5%] vs 123 [37.4%]; *P* < .001), functioning tumors (137 [76.5%] vs 208 [63.2%]; *P* = .008), grade 2 or 3 neuroendocrine tumors (123 [68.7%] vs 164 [49.8%]; *P* < .001), and neuroendocrine tumors with a Ki-67 proliferation index greater than 10% (45 [25.2%] vs 33 [10.0%]; *P* < .001). A higher proportion of patients who received upfront PRRT underwent surgery for the primary tumor (241 [73.3%] vs 96 [53.6%]; *P* < .001). The treatment sequence was completed in 179 patients (100%) in the upfront chemotherapy or targeted therapy group and in 317 patients (96.3%) in the upfront PRRT group (*P* = .01). The mean (SD) number of cycles was higher in the upfront PRRT group compared with the upfront chemotherapy or targeted therapy group (5.0 [0.1] vs 4.2 [0.1]; *P* < .001). In the upfront chemotherapy or targeted therapy group, tandem treatment with yttrium-90 and lutetium-177 was more frequently used (49 patients [27.4%] vs 108 [2.8%]; *P* = .007). The sources of selection bias were the site of tumor origin (SMD, 0.84; standardized bias, 86.4%), functioning tumor status (SMD, 0.29; standardized bias, 30.4%), WHO grade (SMD, 0.38; standardized bias, 85.3%), the Ki-67 proliferation index with a 10% cutoff (SMD, 0.33; standardized bias, 40.3%), surgery for primary tumors (SMD, 0.47; standardized bias, 45.4%), the mean number of PRRT cycles (SMD, 0.49; standardized bias, 50.8%), and the type of radionuclide (SMD, 0.40; standardized bias, 16.3%).

Table 2 and the eTable in the Supplement show the characteristics of the matched groups, which included 222 total patients (mean [SD] age, 56.1 [0.8] years; 124 [55.9%] male), with 111 each in the PRRT arm and the chemotherapy or targeted therapy arm. eFigure 2 in the Supplement shows the standardized bias before and after matching for each variable. eFigure 3 in the Supplement shows the propensity score after and before matching in both groups. All significant differences in unmatched groups were removed (all *P* > .05), obtaining an optimal balance (all standardized bias values were <15%, and all SMDs were <0.20).

**Table 2.** Baseline Characteristics of Matched Populations of Patients With Enteropancreatic Neuroendocrine Tumors Who Received Upfront PRRT or Upfront Chemotherapy or Targeted Therapy

Variable	Patients^a^			*P* value^b^	SMD^c^	Standardized bias, %^d^	Standardized bias reduction after PSM
	Total (N = 222)	Chemotherapy or targeted therapy (n = 111)	PRRT (n = 111)				
Time to progression during first-line SSA therapy, mean (SD), mo	25.1 (2.2)	25.4 (3.2)	25.7 (3.1)	.94	0.01	0.4	96.0
Sex							
Female	98 (44.1)	45 (40.5)	53 (47.8)	>.99	0	14.5	100
Male	124 (55.9)	66 (59.5)	58 (52.3)				
Age, mean (SD), y	56.1 (0.8)	55.9 (1.2)	56.3 (1.1)	.84	0.02	2.7	56.3
ECOG performance status							
≤1	213 (95.9)	106 (95.5)	107 (96.4)	>.99	0.04	4.0	21.7
>2	9 (4.1)	5 (4.5)	4 (3.6)				
Site of tumor origin							
Pancreas	154 (69.4)	73 (65.8)	81 (73.0)	.30	0.15	15.8	81.7
Intestine	68 (30.6)	38 (34.2)	20 (18.0)				
Functioning							
No	159 (71.6)	79 (71.2)	80 (72.1)	>.99	0.02	2.0	93.5
Yes	63 (28.4)	32 (28.8)	31 (27.9)				
MEN1 syndrome							
No	221 (99.6)	110 (99.1)	111 (100)	>.99	0.13	8.5	17.6
Yes	1 (0.4)	1 (0.9)	0				
Grade according to 2019 WHO classification^20^							
1	69 (31.1)	35 (31.5)	34 (30.6)	.57	0.03	3.3	92.7
2	144 (64.9)	70 (63.1)	74 (66.7)				
3	9 (4.1)	6 (5.4)	43 (2.7)				
Ki-67 proliferation index >10%							
No	180 (81.1)	90 (81.1)	90 (81.1)	>.99	0	0	100
Yes	40 (18.0)	20 (18.0)	20 (18.0)				
Data missing	2 (0.9)	1 (0.9)	1 (0.9)				
Surgery for primary tumor							
No	90 (40.5)	42 (37.8)	48 (43.2)	.49	0.11	11.4	74.8
Yes	132 (59.5)	69 (62.2)	63 (56.8)				
Metastases							
Synchronous	191 (86.0)	93 (83.8)	98 (88.3)	.43	0.13	12.7	100
Metachronous	31 (14.0)	18 (16.2)	13 (11.7)				
Surgery for metastases							
No	156 (70.3)	81 (73.0)	75 (67.6)	.36	0.03	0	100
Yes	64 (28.8)	30 (27.0)	34 (30.6)				
Data missing	2 (1.8)	0	2 (1.8)				
Metastatic sites, No.							
1	88 (39.6)	40 (36.0)	48 (43.2)	.30	0.07	6.3	57.1
2	69 (31.1)	39 (35.1)	30 (27.0)				
≥3	51 (23.0)	25 (22.5)	26 (23.4)				
Data missing	14 (6.3)	7 (6.3)	7 (6.3)				
Metastatic sites							
Not reported	14 (6.3)	7 (6.3)	7 (6.3)	.68	0.13	10.9	11.3
Liver	80 (36.0)	36 (32.4)	44 (39.6)				
Liver and extrahepatic	118 (53.2)	62 (55.9)	56 (50.5)				
Extrahepatic	10 (4.5)	6 (5.4)	4 (3.6)				
Hepatic locoregional treatment							
No	162 (73.0)	79 (71.2)	83 (74.8)	.65	0.08	8.1	24.2
Yes	60 (27.0)	32 (28.8)	28 (25.2)				
Sequence completed^e^							
No	0	0	0	>.99	0	0	0
Yes	222 (100)	111 (100)	111 (100)				
SSA during PRRT							
No	27 (12.2)	12 (10.8)	15 (13.5)	.54	0.05	5.3	15.3
Standard dose	194 (87.4)	99 (89.2)	95 (85.6)				
High dose	1 (0.4)	0	1 (0.9)				
Cycles of PRRT, mean (SD), No.	4.7 (0.1)	4.6 (0.2)	4.7 (0.1)				
Radionuclide							
Not reported	4 (1.8)	1 (0.9)	3 (2.7)	.65	0.05	3.2	80.3
Yttrium-90	50 (22.5)	27 (24.3)	23 (20.7)				
Lutetium-177	97 (43.7)	50 (45.1)	47 (42.3)				
Both	71 (32.0)	33 (29.7)	38 (34.2)				

Abbreviations: ECOG, Eastern Cooperative Oncology Group; MEN1, multiple endocrine neoplasm type 1; PRRT, peptide receptor radionuclide therapy; PSM, propensity score matching; SMD, standardized mean difference; SSA, somatostatin analogue; WHO, World Health Organization.

^a^ Data are reported as the number (percentage) of patients unless otherwise indicated.

^b^ The Fisher exact test was used for binary variables, the Pearson χ^2^ test for ordinal variables, and the Student *t* test for continuous variables.

^c^ Effect size categories: small, 0 to 0.2 (nonoverlap population <15%); medium, greater than 0.2 to 0.5 (nonoverlap population <33%); large, greater than 0.5 to 0.8 (nonoverlap population <50%); and very large, greater than 0.8 (nonoverlap population >50%).

^d^ Standardized bias reflects the selection bias as a percentage; a value less than 15% means an optimal balance.

^e^ In the upfront chemotherapy or targeted therapy group, the sequence was considered completed when PRRT was used after chemotherapy or targeted therapy failure; in the upfront PRRT group, the sequence was considered completed when chemotherapy or targeted therapy was used after PRRT failure.

### Survival Analysis

The median follow-up time calculated from the time of diagnosis was 90 months (range, 55.5-131.0 months) in both the unmatched and matched populations. The median follow-up time calculated from disease progression after the first line of somatostatin analogue treatment in the unmatched population was 18 months (range, 8.3-36.0 months) and in the matched population, 13 months (range, 4.9-27.1 months).

Survival outcomes of the unmatched and matched populations are shown in Table 3. In the unmatched population, the median PFS was longer in the upfront PRRT group compared with the upfront chemotherapy or targeted therapy group (2.5 years [95% CI, 2.3-3.0 years] vs 0.7 years [95% CI, 0.5-1.0 years]; HR, 0.35 [95% CI, 0.28-0.44; *P* < .001]) (Figure, A). The use of upfront PRRT was not associated with longer median OS compared with use of upfront chemotherapy or targeted therapy (12.0 years [95% CI, 10.7-14.1 years] vs 11.6 years [95% CI, 9.1-13.4 years]; HR, 0.81 [95% CI, 0.62-1.06; *P* = .11]) (Figure, B).

**Table 3.** Survival Outcomes in Matched and Unmatched Populations of Patients With Enteropancreatic Neuroendocrine Tumors Who Received Upfront PRRT or Upfront Chemotherapy or Targeted Therapy

Outcome	Second-line therapy		HR (95% CI)	*P* value^a^	Third-line therapy		HR (95% CI)	*P* value^a^
	Chemotherapy or targeted therapy, median (95% CI), y	PRRT up front, median (95% CI), y			Chemotherapy or targeted therapy, median (95% CI), y	PRRT, median (95% CI), y		
Unmatched population								
PFS	0.7 (0.5-1.0)	2.5 (2.3-3.0)	0.35 (0.28-0.44)	<.001	0.7 (0.5-0.9)	2.2 (1.9-2.7)	0.26 (0.20-0.35)	<.001
OS	11.6 (9.1-13.4)	12.0 (10.7-14.1)	0.81 (0.62-1.06)	.11	NA	NA	NA	NA
Matched population								
PFS	0.6 (0.4-1.0)	2.2 (1.8-2.8)	0.37 (0.27-0.51)	<.001	1.0 (0.5-1.2)	2.2 (1.9-2.7)	0.31 (0.21-0.45)	<.001
OS	11.5 (9.2-17.9)	12.2 (9.1-14.2)	0.83 (0.56-1.24)	.36	NA	NA	NA	NA

Abbreviations: HR, hazard ratio; NA, not applicable; OS, overall survival; PFS, progression-free survival; PRRT, peptide receptor radionuclide therapy.

^a^ From the Cox proportional hazards regression analysis.

**Figure.**
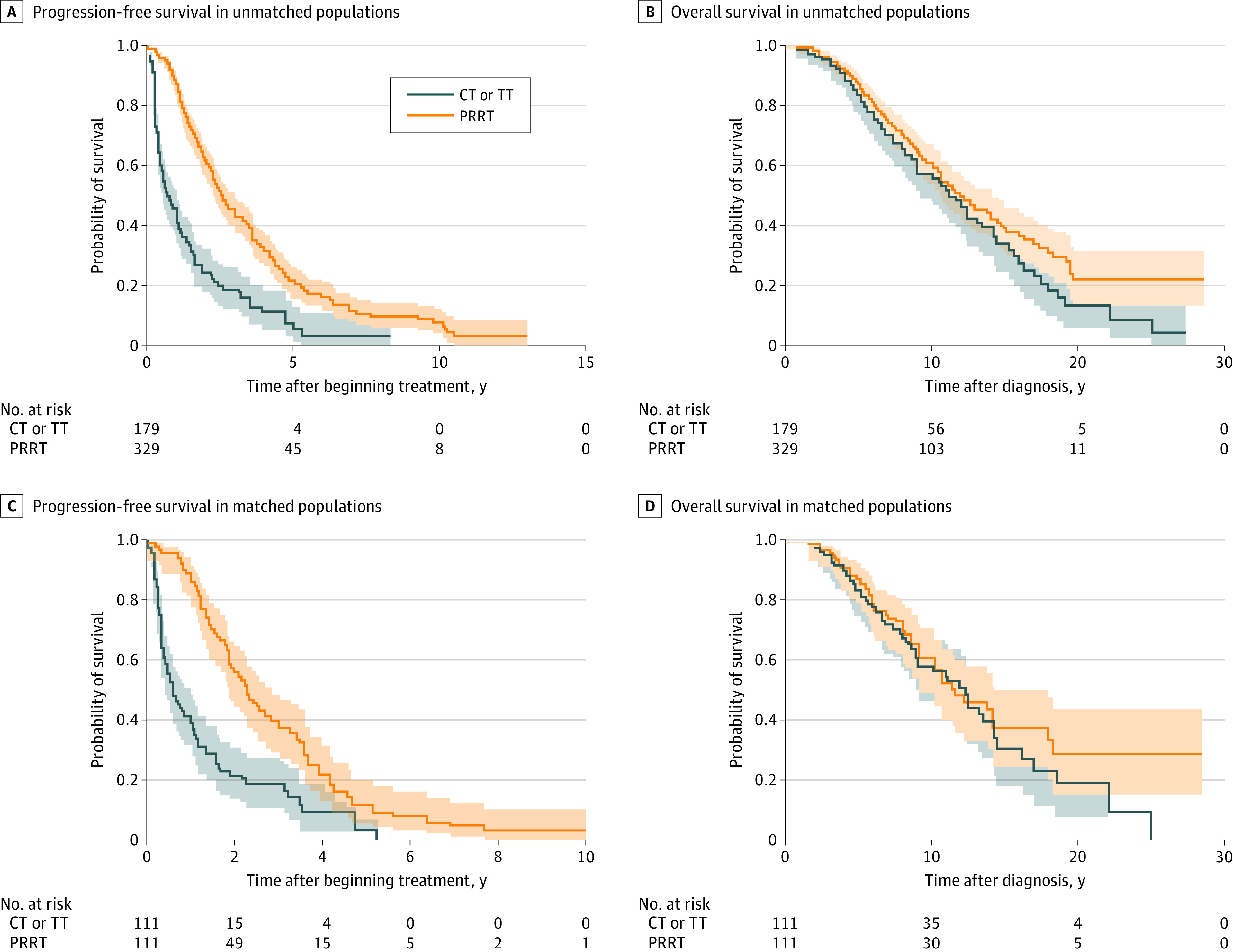
Progression-Free Survival and Overall Survival in Unmatched and Matched Populations of Patients With Enteropancreatic Neuroendocrine Tumors Who Received Upfront Peptide Receptor Radionuclide Therapy (PRRT) or Upfront Chemotherapy (CT) or Targeted Therapy (TT) Shaded areas represent X.

In the matched population, patients in the upfront PRRT group had longer median PFS compared with those in the upfront chemotherapy or targeted therapy group (2.2 years [95% CI, 1.8-2.8 years] vs 0.6 years [95% CI, 0.4-1.0 years]; HR, 0.37 [95% CI, 0.27-0.51; *P* < .001]) (Figure, C). The OS analysis in the matched group showed that upfront PRRT was associated with a median OS 8.4 months longer compared with upfront chemotherapy or targeted therapy (12.2 years [95% CI, 9.1-14.2 years] vs 11.5 years [95% CI, 9.2-17.9 years]; HR, 0.83 [95% CI, 0.56-1.24; *P* = .36]) (Figure, D).

### Multivariable Analysis

In multivariable analysis (Table 4), the use of upfront PRRT was independently associated with longer PFS (HR, 0.37; 95% CI, 0.26-0.51; *P* < .001). Grade 3 tumors were also independently associated with PFS (HR, 2.64; 95% CI, 1.19-6.27; *P* = .01). Tumor functional status, the primary tumor site, and the Ki-67 proliferation index were not associated with PFS.

**Table 4.** Multivariate Model for Progression-Free Survival in the Matched Population of Patients With Enteropancreatic Neuroendocrine Tumors Who Received Upfront PRRT or Upfront Chemotherapy or Targeted Therapy

Covariate	HR (95% CI)	*P* value	Interaction between PRRT and other covariates	
			aHR (95% CI)	*P* value
Treatment group				
Chemotherapy or targeted therapy	1 [Reference]	<.001	NA	NA
PRRT	0.37 (0.26-0.51)		NA	NA
Functioning tumors				
No	1 [Reference]	.67	0.29 (0.16-0.56)	<.001
Yes	0.91 (0.66-1.31)		0.39 (0.27-0.57)	<.001
Primary site				
Pancreas	1 [Reference]	.12	0.41 (0.24-0.61)	<.001
Intestine	0.97 (0.71-1.34)		0.19 (0.11-0.43)	<.001
Grade according to 2019 WHO classification^20^				
1	1 [Reference]	NA	0.21 (0.12-0.34)	<.001
2	0.95 (0.67-1.36)	.84	0.52 (0.29-0.73)	<.001
3	2.64 (1.19-6.27)	.01	0.31 (0.12-1.37)	.13
Ki-67 proliferation index >10%				
No	1 [Reference]	.47	0.71 (0.18-0.37)	<.001
Yes	0.86 (0.56-1.31)		0.73 (0.29-1.43)	.31

Abbreviations: aHR, adjusted hazard ratio; HR, hazard ratio; NA, not applicable; PRRT, peptide receptor radionuclide therapy; WHO, World Health Organization.

The interaction between PRRT and other covariates was significant (Table 4). The HRs adjusted for the interaction showed improved PFS associated with the use of upfront PRRT that was consistent across several subgroups of patients stratified according to different factors such as functioning tumor (adjusted HR [aHR], 0.39; 95% CI, 0.27-0.57; *P* < .001) vs nonfunctioning tumor (aHR, 0.29; 95% CI, 0.16-0.56; *P* < .001); pancreatic tumor (aHR, 0.41; 95% CI, 0.24-0.61; *P* < .001; absolute PFS difference, 1.6 years) vs intestinal tumor (aHR, 0.19; 95% CI, 0.11-0.43; *P* < .001); grade 1 tumor (aHR, 0.21; 95% CI, 0.12-0.34; *P* < .001) vs grade 2 tumor (aHR, 0.52; 95% CI, 0.29-0.73; *P* < .001); and a Ki-67 proliferation index of 10% or less (aHR, 0.71; 95% CI, 0.18-0.37; *P* < .001).

Conversely, there was no association of the upfront PRRT approach with improved PFS in the grade 3 neuroendocrine tumor subgroup (aHR, 0.31; 95% CI, 0.12-1.37; *P* = .13) or in the group with tumors with a Ki-67 proliferation index greater than 10% (aHR, 0.73; 95% CI, 0.29-1.43; *P* = .31).

## Discussion

In this multicenter, retrospective cohort study of 508 patients with advanced enteropancreatic neuroendocrine tumors, we observed that use of upfront PRRT immediately after disease progression with SSA treatment was associated with improved PFS outcomes compared with upfront chemotherapy or targeted therapy. This improvement was consistent in the subgroups with functioning and nonfunctioning neuroendocrine tumors, pancreatic and intestinal neuroendocrine tumors, and grade 1 and 2 tumors (with a Ki-67 proliferation index ≤10%).

After propensity score matching, the multivariable analysis supported these data, showing an independent association between PFS and upfront PRRT after adjusting for other factors, such as the primary tumor site, functional status, tumor grade, and Ki-67 proliferation index. The adjustment of HRs for interaction suggested that use of upfront PRRT was significantly associated with longer PFS regardless of the primary tumor site, functional or nonfunctional tumor status, and tumor grade (grade 1 or 2 tumors with a Ki-67 proliferation index <10%).

Furthermore, in the subgroup of patients with pancreatic neuroendocrine tumors, the aHR for PFS was 0.41 (95% CI, 0.24-0.61), with an absolute difference of 1.6 years in favor of PRRT, suggesting that also in pancreatic neuroendocrine tumors, an appropriate early use of PRRT after disease progression after SSA treatment may be associated with longer PFS. Prospective trials are, however, required to further investigate this finding.

A phase 3, multicenter, randomized, open-label trial is currently ongoing to determine whether first-line treatment with PRRT (^177^Lu-dotatate in combination with long-acting octreotide) prolongs PFS in patients with gastroenteropancreatic neuroendocrine tumors with highly proliferating tumors (grades 2 and 3) compared with high-dose long-acting octreotide (NETTER-2 trial).^21^ Another trial is ongoing to evaluate the efficacy and safety of the targeted radiopharmaceutical ^177^Lu-edotreotide compared with everolimus in patients with gastroenteropancreatic neuroendocrine tumors (COMPETE trial).^22^

Although some studies^18,20,23^ have reported favorable survival rates associated with PRRT for well-differentiated neuroendocrine tumors, the current study did not detect an improvement in OS associated with use of upfront PRRT. Although a clinically meaningful improvement in median OS (8.4 months) was reported in patients receiving upfront PRRT, the analysis in the unmatched and matched populations did not show a reduction in the risk of death compared with patients receiving upfront chemotherapy or targeted therapy. However, in a retrospective study with a long observation period on a relatively indolent disease, the use of OS as a primary or secondary endpoint is particularly challenging because of extended survival and the use of a range of salvage therapies after disease progression. In addition, because the patients in our study were recruited during a prolonged period (2000-2020), we cannot exclude that the improvement in care over time, associated with the heterogeneity and different availability of treatments between the participating oncology centers, also may have affected the final OS results.

Our data are consistent with the final OS results reported in the randomized phase 3 NETTER-1 clinical trial.^23^ In that trial, PRRT was associated with a survival benefit of 11.7 months (HR, 0.84; 95% CI, 0.6-1.1; *P* = .30), although this finding did not reach significance because the crossover of patients likely confounded the OS results. Similarly, in our study, 2 therapeutic sequences (chemotherapy or targeted therapy followed by PRRT vs PRRT followed by chemotherapy or targeted therapy) were compared, in which the high crossover rate (100%) of patients in the control group who received radioligand therapy after progression to chemotherapy or targeted therapy may also have also been associated with improved OS outcomes. A prospective, multicenter, controlled, randomized, open-label clinical trial should be encouraged with the aim to overcome these limitations and to collect data with greater relevance for clinical practice.

### Limitations

This study has limitations. The main limitation of our analysis is its retrospective design, which does not provide the strength of evidence of a randomized comparison, especially considering that the 2 groups differed significantly with regard to several clinical characteristics in the unmatched population. Furthermore, some information (eg, tumor size) was not properly collected owing to the retrospective nature of the study and therefore was not included in our analysis. However, the use of propensity score matching in both groups removed all differences in the unmatched population, balancing the groups and reinforcing the validity of our findings. Data on safety were also not included in the present study because they will be more extensively and comprehensively analyzed in a future study.

## Conclusions

This cohort study assessed for the first time, to our knowledge, a real-world population of patients with advanced enteropancreatic neuroendocrine tumors who received approved systemic therapies for advanced disease (PRRT, SSAs, everolimus, sunitinib, and chemotherapy). Patients who received upfront PRRT after experiencing disease progression with SSA treatment and who had a Ki-67 proliferation index of 10% or less had statistically and clinically meaningfully prolonged PFS compared with patients who received upfront chemotherapy or targeted therapy. Standardization of clinical practice into a well-defined therapeutic algorithm may be challenging, and prospective randomized phase 3 clinical trials are needed to further investigate the correct strategy, timing, and optimal specific sequence of these therapeutic options. In addition, phase 4 trials are warranted to gain further insights into the potential adverse effects that can occur with the long-term use of these treatments also in correlation with different therapeutic sequences.

## Notes

Supplement. **eTable.** Distribution of Patients by Treatments (Chemotherapy or Targeted Therapy) Among Patients in Unmatched and Matched Population **eFigure 1.** Multicenter, Retrospective, Italian (It.a.net) Cohort Study Design **eFigure 2.** Standardized Bias Before and After Propensity Score Matching for All Variables **eFigure 3.** Kernel Density Plot
