# Additional Information Section Added

**DOI:** 10.1001/jamanetworkopen.2022.7517

**Published:** 2022-03-24

In the Original Investigation titled “Association of Upfront Peptide Receptor Radionuclide Therapy With Progression-Free Survival Among Patients With Enteropancreatic Neuroendocrine Tumors,”^1^ published February 24, 2022, an Additional Information section was added to indicate that the study was endorsed by the Italian Association for Neuroendocrine Tumors (It.a.net). This article has been corrected.^1^
