# Supplementary material for: Association of Upfront Peptide Receptor Radionuclide Therapy With Progression-Free Survival Among Patients With Enteropancreatic Neuroendocrine Tumors
Source: JAMA Netw Open. 2022 Feb 24;5(2):e220290. doi: 10.1001/jamanetworkopen.2022.0290 (PMC8874344; doi:10.1001/jamanetworkopen.2022.0290)
Supplement: Supplement. — eTable. Distribution of Patients by Treatments (Chemotherapy or Targeted Therapy) Among Patients in Unmatched and Matched Population eFigure 1. Multicenter, Retrospective, Italian (It.a.net) Cohort Study Design eFigure 2. Standardized Bias Before and After Propensity Score Matching for All Variables eFigure 3. Kernel Density Plot [file jamanetwopen-e220290-s001.pdf]

# Supplementary Online Content

Pusceddu S, Prinzi N, Tafuto S, et al. Association of upfront peptide receptor radionuclide therapy with progression-free survival among patients with enteropancreatic neuroendocrine tumors. *JAMA Netw Open*. 2022;5(2):e220290.
doi:10.1001/jamanetworkopen.2022.0290

**eTable.** Distribution of Patients by Treatments (Chemotherapy or Targeted Therapy) Among Patients in Unmatched and Matched Population
**eFigure 1.** Multicenter, Retrospective, Italian (It.a.net) Cohort Study Design
**eFigure 2.** Standardized Bias Before and After Propensity Score Matching for All Variables
**eFigure 3.** Kernel Density Plot

This supplementary material has been provided by the authors to give readers additional information about their work.

© 2022 Pusceddu S et al. *JAMA Network Open*.

**Supplemental Material**

**eTable.** Distribution of patients by treatments (Chemotherapy or Targeted Therapy) among patients in unmatched and matched population

**eFigure 1.** Multicenter, Retrospective, Italian (It.a.net) cohort study design

**eFigure 2**. Standardized bias before and after propensity score matching for all variables

**eFigure 3.** Kernel density plot. The curves represent the distribution of propensity score among the two groups **(A)** before the matching and **(B)** after the matching

© 2022 Pusceddu S et al. *JAMA Network Open*.

**eTable.** Distribution of patients by treatments (Chemotherapy or Targeted Therapy) among patients in unmatched and matched population.

| | **Total, n (%)** | **CT/TT up-front n(%)** | **PRRT up-front n(%)** |
|---|---|---|---|
| *Unmatched population CT /TT (upfront- Second line) (n=179)* | | | |
| Chemotherapy | 121 (67.6) | | |
| • CDDP or OHP based | - | 74 (61.2) | - |
| • TMZ based | - | 12 (9.9) | - |
| • 5-FU based | - | 15 (12.4) | - |
| • Unknown | - | 20 (16.5) | - |
| Targeted Therapy | 58 (32.4) | | |
| • Everolimus | - | 55 (94.8) | - |
| • Sunitinib | - | 3 (5.2) | - |
| *Unmatched population CT /TT (Third line) (n= 116)* | | | |
| Chemotherapy | 52 (44.8) | | |
| • CDDP or OHP based | - | - | 14 (26.9) |
| • TMZ based | - | - | 21 (40.4) |
| • 5-FU based | - | - | 12 (23.1) |
| • Unknown | - | - | 5 (9.6) |
| Targeted therapy | 64 (55.2) | | |
| • Everolimus | - | - | 53 (82.8) |
| • Sunitinib | - | - | 11 (17.2) |
| *Matched population CT /TT (upfront- Second line) (n=111)* | | | |
| Chemotherapy | 72 (64.9) | | |
| CDDP or OHP based | - | 43 (59.7) | - |
| TMZ based | - | 10 (13.9) | - |
| 5-FU based | - | 12 (16.7) | - |
| Unknown | - | 7 (9.7) | - |
| Targeted therapy | 39 (35.1) | | |
| Everolimus | - | 36 (92.3) | - |
| Sunitinib | - | 3 (7.7) | - |
| *Matched population CT /TT (Third line)* | | | |

© 2022 Pusceddu S et al. *JAMA Network Open*.

| (n=25) | | | |
|---|---|---|---|
| Chemotherapy | 10 (40%) | | |
| CDDP or OHP based | - | - | 1 (20) |
| TMZ based | - | - | 8 (80) |
| 5-FU | - | - | 1 (20) |
| Targeted Therapy | 15 (60%) | | |
| Everolimus | - | - | 12 (80) |
| Sunitinib | - | - | 3 (20) |

CT: Chemotherapy, TT: target therapy including treatment with everolimus or sunitinib; PRRT: peptide receptor radionuclide therapy; TMZ: Temozolomide; OHP: Oxaliplatin; 5FU: 5-Fluorouracil.

© 2022 Pusceddu S et al. *JAMA Network Open*.

**eFigure 1.** Multicenter, Retrospective, Italian (It.a.net) cohort study. Study design.

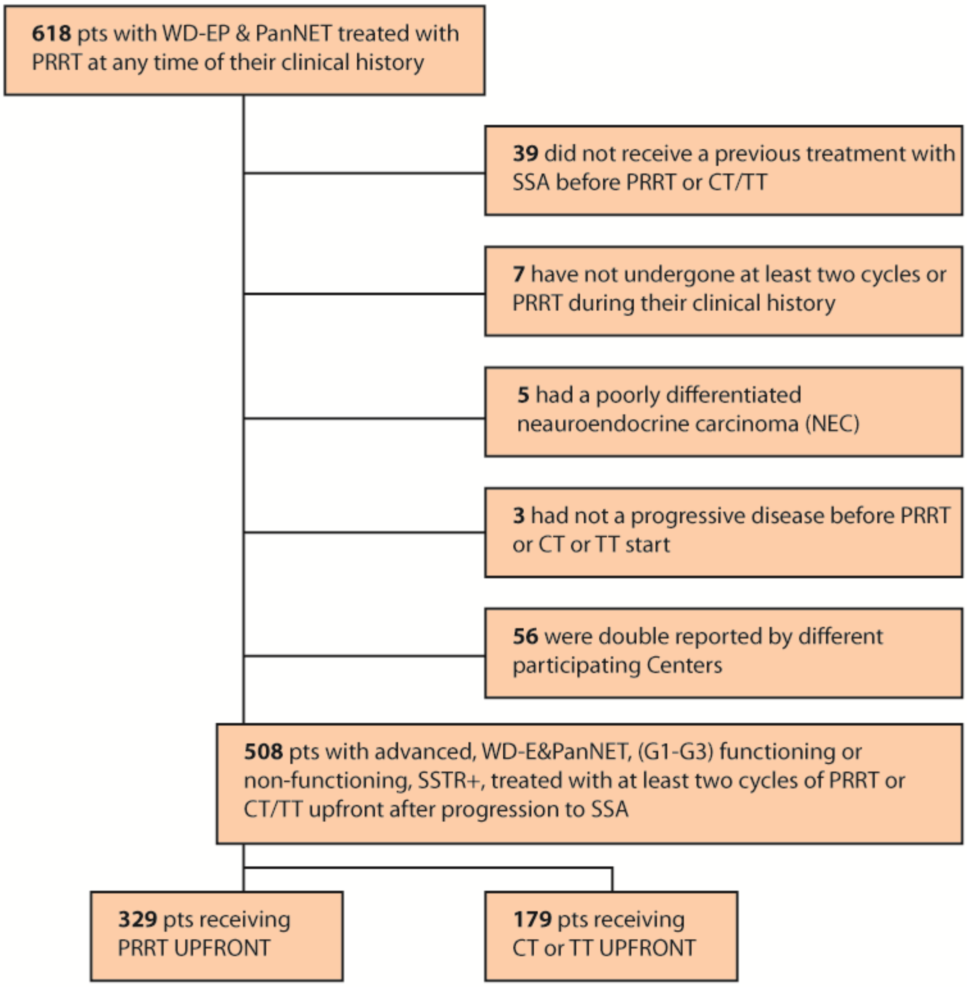


WD: Well-differentiated: E-P entero-pancreatic; PRRT: Peptide Receptor Radionuclide Therapy; NET: neuroendocrine tumor; SSTR+: Somatostatic Receptors positivity; SSA: somatostatin analogues; CT: chemothearpy; TT: Targeted Therapy

© 2022 Pusceddu S et al. *JAMA Network Open*.

**eFigure 2**. Standardized bias before and after propensity score matching for all variables**.**

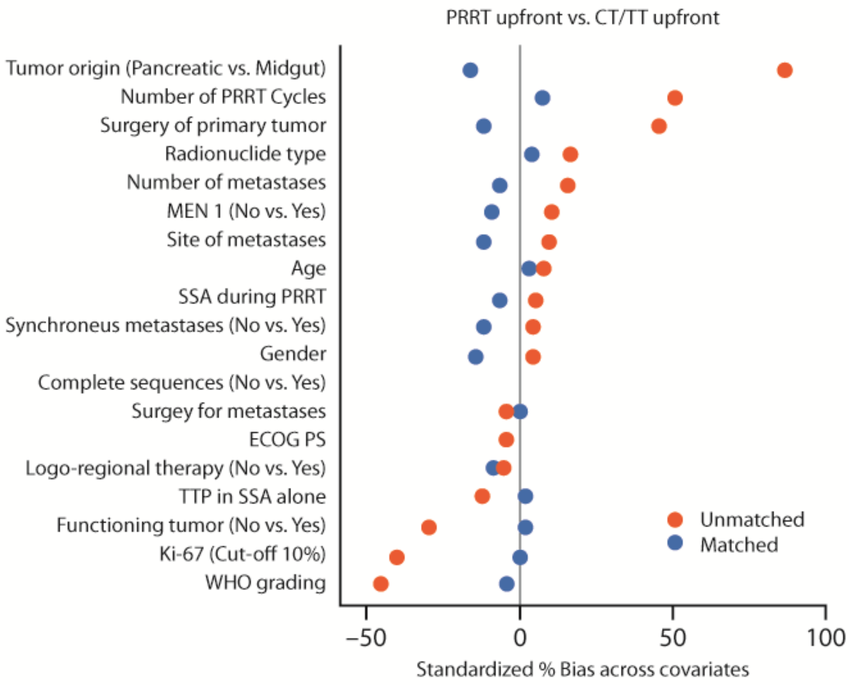


© 2022 Pusceddu S et al. *JAMA Network Open*.

**eFigure 3.** Kernel density plot. The curves represent the distribution of propensity score among the two groups **(A)** before the matching and **(B)** after the matching.

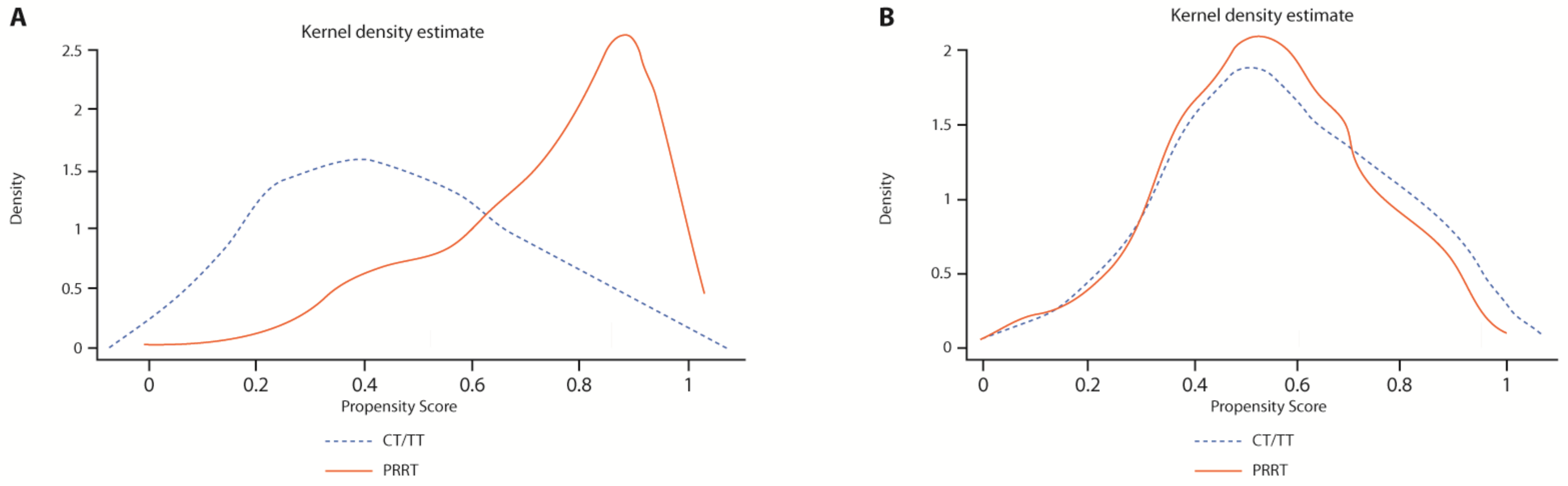


© 2022 Pusceddu S  et al. *JAMA Network Open*.
